# The Role of Vitamin D in Retinal Physiology

**DOI:** 10.22336/rjo.2025.51

**Published:** 2025

**Authors:** Ana-Maria Stoica, Sanda Jurja, Nejla Dervis

**Affiliations:** 1 Department of Ophthalmology, “Sf. Apostol Andrei” County Emergency Clinical Hospital, Constanţa, Romania; 2 Department of Ophthalmology, General Medicine Faculty, Ovidius University, Constanţa, Romania; 3 Department of Diabetes, “Sf. Apostol Andrei” County Emergency Clinical Hospital, Constanţa, Romania

**Keywords:** vitamin D, retina, diabetic retinopathy, AMD, AMD = Age-Related Macular Degeneration, DR = Diabetic Retinopathy, VDR = Vitamin D Receptor, RPE = Retinal Pigment Epithelium, EC = Endothelial Cells, NPDR = Non-Proliferative Diabetic Retinopathy, PDR = Proliferative Diabetic Retinopathy, UVB = Ultraviolet B, IU = International Units, RCT = Randomized Controlled Trial, NMDA = N-Methyl-D-Aspartate, AMPK = AMP-activated protein kinase, TGF = transforming growth factor, EGF = epidermal growth factor, RGCs = retinal ganglion cells, RXRα = retinoid X receptor, VDREs = vitamin D response elements

## Abstract

Vitamin D plays a crucial role in ocular health, particularly in the function and protection of the retina. This fat-soluble vitamin is synthesized in the skin in response to UVB radiation and can also be obtained from dietary sources. Research indicates that vitamin D has neuroprotective properties, which are essential for retinal cell survival and function. The active form of vitamin D, calcitriol, has been shown to modulate inflammation and oxidative stress in retinal tissues, thus potentially preventing retinal degeneration diseases such as age-related macular degeneration (AMD) and diabetic retinopathy. Furthermore, vitamin D receptors are expressed in various retinal cells, suggesting that vitamin D directly influences retinal physiology. Deficiency in vitamin D has been associated with an increased risk of chronic eye diseases, emphasizing the importance of maintaining adequate vitamin D levels for preserving retinal health. Ongoing studies are needed to elucidate further the molecular mechanisms underlying the protective effects of vitamin D on the retina and to explore its therapeutic potential in retinal disorders.

## Introduction

Vitamin D, a secosteroid with hormonal action, is widely recognized for its essential role in maintaining bone health and mineral balance. However, research over recent decades has revealed a multitude of underestimated extra-skeletal benefits, underscoring its broader systemic importance. These include vital functions in brain health, modulation of the immune system, pregnancy and birth outcomes, cancer prevention, and cardiovascular integrity. Its biological action is mediated primarily through binding to the Vitamin D Receptor (VDR), a key protein of the nuclear hormone receptor superfamily, which regulates fundamental cellular processes such as proliferation, differentiation, apoptosis, and both adaptive and innate immunity [1].

Clinically, maintaining optimal serum concentrations of 25(OH)D is crucial; levels above 30 ng/mL (75 nmol/L) are associated with a significant reduction in disease risks and mortality, while concentrations between 40 and 70 ng/mL may offer enhanced protection against many adverse health outcomes. In addition, vitamin D acts as a powerful antioxidant, an immunomodulator, and a regulator of angiogenesis, directly or indirectly influencing the expression of more than 900 genes in the human genome [2,3].

The retina is a complex and highly specialized neural tissue located in the posterior segment of the eye, representing a direct extension of the brain. Its primary function is to capture light photons and transform them into bioelectrical signals, which are then transmitted to the brain for visual perception. This tissue is organized into multiple distinct layers, each containing specialized cell types, including photoreceptors (rods and cones), the retinal pigment epithelium (RPE), bipolar cells, horizontal cells, amacrine cells, ganglion cells, and supportive Müller glial cells [4].

Photoreceptor cells, particularly their outer segments, contain photosensitive pigments such as rhodopsin, which are essential for phototransduction and exhibit exceptionally high metabolic demands. The RPE, the outermost layer of the retina, is vital for supporting photoreceptors, maintaining overall retinal homeostasis, and significantly contributing to the integrity of the blood-retina barrier [4,5].

It is essential to emphasize the distinction between the roles of vitamin D and vitamin A. While vitamin A (in the form of retinal) is an indispensable dietary component for the synthesis of rhodopsin and for facilitating the phototransduction pathway, vitamin D’s influence on retinal physiology is distinct, encompassing broader cellular regulation, protective mechanisms, and modulation of pathological processes [4].

A growing body of evidence indicates a significant and direct influence of vitamin D on ocular health, particularly within the retina, extending beyond its well-established systemic effects. This influence is supported by the documented presence of vitamin D receptors and metabolizing enzymes in various ocular tissues. This report will explore in detail the specific molecular mechanisms and significant clinical implications of vitamin D’s role in maintaining the physiological integrity of the retina and its associations with the pathogenesis and progression of various retinal disorders [6].

## Vitamin D Signaling in the Retina: Local Metabolism and Molecular Mechanisms

The classic pathway of systemic vitamin D activation involves a two-step hydroxylation process. Initially, vitamin D (either D3 or D2) undergoes 25-hydroxylation in the liver, resulting in 25-hydroxyvitamin D (25(OH)D), which serves as the main circulating form and biomarker of vitamin D status. Subsequently, 25(OH)D is converted into its biologically active form, 1,25-dihydroxyvitamin D (1,25(OH)_2_D), through the action of the enzyme 1α-hydroxylase (encoded by the CYP27B1 gene), primarily in the kidney [6].

The biological action of vitamin D depends on the presence of the Vitamin D Receptor (VDR) in target tissues. Compelling evidence indicates that VDRs are widely expressed in various critical retinal tissues, including the retinal pigment epithelium (RPE), photoreceptors, and the complex retinal vascular network. More specifically, VDR expression has been clearly demonstrated in retinal endothelial cells. In the adult mouse retina, VDR expression is precisely localized in the proximal inner nuclear layer and in the ganglion cell layer. This specific distribution of VDR in key retinal cells, such as RPE cells, photoreceptors, vascular cells, ganglion cells, and cells in the inner nuclear layer, provides a direct biological basis for the local action of vitamin D in the retina [7].

This means that the retina is not only a tissue influenced by systemic vitamin D levels but also a direct target tissue capable of responding to local vitamin D signaling. Notably, VDR expression in retinal endothelial cells is induced by calcitriol, the active form of vitamin D. This observation suggests a dynamic regulatory feedback loop in which the presence of active vitamin D can amplify the tissue’s response to subsequent vitamin D signaling [7,8].

Beyond the mere presence of VDR, ocular tissues -including the retina- possess the intrinsic enzymatic machinery needed to activate and regulate vitamin D locally. Studies have confirmed measurable levels of both the precursor 25(OH)D_3_ and the active form 1,25(OH)_2_D_3_ in the aqueous and vitreous humors of the eye. This indicates that the active form of vitamin D is available in the ocular environment. Moreover, the enzyme 1α-hydroxylase (CYP27B1), responsible for the critical conversion of 25(OH)D to its active form, is expressed in ocular tissues. Direct evidence from the Human Protein Atlas also confirms CYP27B1 mRNA expression in the human retina, with a normalized average value of 3.2 nTPM (Tags Per Million) in various samples. This concrete molecular evidence of local activation capacity in the retina suggests the existence of a localized, autocrine or paracrine regulatory system within the eye [6].

This allows for fine control of vitamin D signaling, which can operate, to some extent, independently of systemic vitamin D levels, enabling the retina to modulate its own vitamin D responses in a context-specific manner [9,10].

Vitamin D exerts its functions primarily by binding to VDR, which then acts as a ligand-modulated nuclear receptor to influence the expression of numerous target genes. This VDR engagement plays a pivotal role in regulating genes involved in crucial processes such as angiogenesis and inflammation. VDR can also interact with other nuclear transcription factors, particularly the retinoid X receptor (RXRα). This complex then binds to specific vitamin D response elements (VDREs) in the promoters of target genes, leading to either activation or transcriptional repression [8].

Beyond these classical transcriptional effects, 1,25(OH)_2_D_3_ has been shown to modulate rapid, non-genomic signaling pathways triggered at the plasma membrane by various agents, including Wnt, transforming growth factor (TGF)-β, and epidermal growth factor (EGF). In addition, vitamin D can influence gene expression through paracrine mechanisms, regulating cytokine or growth factor secretion by neighboring cells. It also exerts post-transcriptional and post-translational effects that can amplify or overlap with its classical modulation of gene transcription rates [11].

In the context of metabolic processes, vitamin D is known to modulate pro-inflammatory cytokines, activate the AMPK (AMP-activated protein kinase) pathway, and inhibit mTOR (mammalian target of rapamycin) signaling. These pathways are critical for cellular energy balance, growth, and inflammation [12].

This multilayered molecular action - encompassing not only direct gene regulation but also interactions with membrane-based signaling pathways, paracrine mechanisms, and modulation of key metabolic regulators - enables vitamin D to exert diverse and profound effects on cell function, differentiation, and survival within the highly complex retinal environment [6].

## The Multifunctional Roles of Vitamin D in Retinal Homeostasis and Protection

Vitamin D is widely recognized as a potent immunomodulator and anti-inflammatory agent, playing a crucial role in regulating immune responses. In the context of retinal health, studies have demonstrated its ability to suppress oxidative damage and inflammation specifically in retinal pigment epithelial (RPE) cells. When RPE cells were subjected to oxidative stress, vitamin D treatment significantly increased cell viability by activating antioxidant signaling pathways. Animal studies further support this, showing that vitamin D supplementation leads to a notable reduction in macrophage numbers, thereby alleviating retinal inflammation [13]. The consistent observation of vitamin D’s anti-inflammatory effects in various retinal cell types and its demonstrated ability to reduce overall retinal inflammation in animal models strongly suggests that inflammation is a central pathological process in many retinal disorders. Vitamin D’s ability to consistently target this fundamental mechanism positions it as a key endogenous protective factor [14].

Vitamin D also functions as a robust antioxidant, protecting retinal cells against the harmful effects of oxidative stress. Oxidative stress is a well-established critical factor in the initiation and progression of numerous retinal disorders, including age-related macular degeneration (AMD) and diabetic retinopathy (DR). The demonstrated ability of vitamin D to mitigate oxidative damage in RPE cells directly underscores its protective role against a significant underlying factor of retinal pathology and cellular dysfunction [8].

Given the retina’s inherently high metabolic rate and its constant exposure to light, oxidative stress represents a continuous and significant threat to cellular integrity. Vitamin D’s direct antioxidant action provides a crucial endogenous defense mechanism, safeguarding the health, structure, and function of retinal cells against this pervasive challenge [13].

The structural and functional integrity of retinal endothelial cells (ECs) is paramount for establishing and maintaining the blood-retina barrier, which is essential for proper vision. Vitamin D, primarily through its interaction with VDR, plays a protective role in EC function by regulating the expression of genes involved in both angiogenesis (the formation of new blood vessels) and inflammation [14].

Calcitriol, the active form of vitamin D, has been identified as a potent inhibitor of retinal neovascularization in vivo (in mouse models of oxygen-induced ischemic retinopathy) and of capillary morphogenesis of retinal ECs in vitro. A deficiency in VDR expression significantly affects EC-EC and EC-matrix interactions, leading to slower proliferation rates, increased adhesion, and reduced migratory capacity of retinal ECs. Furthermore, VDR-deficient retinal ECs show increased expression of inflammatory markers and heightened sensitivity to oxidative challenges [10,14].

Vitamin D also shows inhibitory strength against key pro-angiogenic factors, such as retinal VEGF (vascular endothelial growth factor) and transforming growth factor. VDR expression is crucial during retinal vascular development, contributing to the maturation of the retinal vasculature by promoting pericyte quiescence and endothelial cell survival, while inhibiting ischemia-mediated retinal neovascularization [8,15].

Vitamin D’s role extends beyond simply inhibiting pathological angiogenesis; it acts as a comprehensive regulator of retinal vascular homeostasis. Its multifunctional involvement in maintaining EC integrity, modulating cell-cell interactions, and suppressing pathological neovascularization is critically essential for preventing and mitigating conditions such as diabetic retinopathy, where vascular dysfunction is a central pathological feature [8,10,14].

Vitamin D has also been increasingly recognized for its significant neuroprotective properties. In the context of vision, photoreceptor death is an unavoidable cause of blindness, and neuroprotection strategies are actively pursued to delay photoreceptor degeneration and preserve visual function [16].

Experimental studies have provided direct evidence that vitamin D_3_ exerts a neuroprotective effect on the thickness of the ganglion cell-inner plexiform layer in models of NMDA-induced excitotoxic glaucoma, thus protecting retinal ganglion cells (RGCs). This suggests a direct impact on the survival of retinal neurons responsible for transmitting visual information to the brain [17].

Animal research also suggests that vitamin D may help protect the retina from the harmful effects of aging and help maintain its optimal function. This protective effect may be partially mediated through the reduction of intracellular beta-amyloid accumulation, a known aging biomarker in retinal cells [13].

Beyond its supportive roles for the retinal pigment epithelium and vasculature, vitamin D directly contributes to the survival and health of retinal neurons, particularly retinal ganglion cells and potentially photoreceptors, protecting them from degeneration. This highlights a crucial role in preserving both the neural integrity and the functional outcome of the retina itself [13].

## Clinical Implications: Vitamin D in Retinal Diseases

### Diabetic Retinopathy (DR)

Diabetic retinopathy (DR) is a severe microvascular complication of diabetes mellitus (DM) and remains a leading global cause of visual impairment and blindness, resulting from chronic hyperglycemia-induced damage to retinal blood vessels. Vitamin D deficiency (VDD) is also highly prevalent among patients with type 2 diabetes [8].

A substantial body of evidence, including multiple systematic reviews and meta-analyses, consistently reports a significant inverse association between low vitamin D levels and an increased risk or severity of DR. For example, a meta-analysis encompassing 20 studies and 22,408 participants found a significant association between low vitamin D levels and an increased likelihood of DR, with a combined odds ratio (OR) of 1.17 [18].

This analysis showed that the mean serum vitamin D levels were consistently lower in individuals with DR (18.11±5.35 ng/mL) compared to those without DR (19.71±7.44 ng/mL), with a progressive decrease observed as DR severity increased. Moreover, significantly lower vitamin D levels were explicitly noted in cases of proliferative DR (the more advanced stage) compared to non-proliferative stages [18].

The proposed biological mechanisms linking VDD to DR pathogenesis are multifactorial, involving primarily vitamin D’s effects on inflammation, oxidative stress, and vascular function. The established anti-inflammatory, antioxidant, and anti-angiogenic properties of vitamin D are crucial for preserving the integrity and healthy function of the retinal vasculature, which is compromised in DR [10,14].

Regarding the impact of supplementation, a retrospective cohort study focusing on newly diagnosed type 2 diabetes patients found that VDD (defined as < 20 ng/mL) was significantly associated with an increased risk of developing DR (Hazard Ratio 1.45) over a 3-year follow-up period, with this association being particularly pronounced in female patients [1,8].

This study also provided a crucial finding, suggesting a “threshold effect”: vitamin D insufficiency (levels between 20-30 ng/mL) was not associated with an increased risk of DR, implying that maintaining vitamin D levels above the deficiency threshold (i.e., ≥ 20 ng/mL) might be sufficient to mitigate the risk of DR progression [18].

A smaller prospective interventional study (40 patients) demonstrated that daily oral vitamin D supplementation (2000 IU/day for 6 months), added to standard photocoagulation treatment, significantly delayed the progression of severe and very severe non-proliferative DR (NPDR) and early proliferative DR (PDR) [18].

This study also reaffirmed the inverse relationship between serum 25(OH)D levels and DR severity, suggesting that low vitamin D levels might serve as a predictor for DR severity.

The abundance and consistency of observational evidence, coupled with promising preliminary interventional study results, firmly position diabetic retinopathy as a primary clinical target for vitamin D intervention. The identification of a specific “threshold effect” (below 20 ng/mL) offers practical and actionable guidance for clinical management, suggesting that preventing severe vitamin D deficiency may be a key strategy in mitigating DR risk and progression [19].

### Age-Related Macular Degeneration (AMD)

Observational studies have historically suggested a potential association between higher dietary intake or higher blood levels of vitamin D and a lower risk of age-related macular degeneration (AMD). This biological plausibility is supported by the hypothesized protective effects of vitamin D, including reducing oxidative stress, inflammation, and angiogenesis - all central processes in AMD pathogenesis [14].

However, evidence from large-scale randomized controlled trials (RCTs) is more nuanced. The VITAL study, a prominent RCT involving 25,871 participants, specifically evaluated the effect of daily vitamin D_3_ supplementation (2000 IU/day) over a median follow-up period of 5.3 years [14].

This study found no overall significant effect on AMD incidence or progression (Hazard Ratio 1.02 for total AMD events, with similarly null results for AMD incidence and progression components) [14].

The stark contradiction between observational findings (suggesting an association) and the null results from a large, well-conducted randomized controlled trial (RCT), as observed in the VITAL study, on AMD incidence and progression, is a crucial finding. This discrepancy highlights the inherent challenges in translating observed correlations into definitive causal or therapeutic effects, underscoring the critical importance of robust clinical trial data in informing clinical practice [20].

## Other Ocular Conditions with Retinal Relevance

Recent research indicates a potential link between vitamin D deficiency and uveitis relapses, a significant inflammatory ocular condition. Studies have shown that patients with active uveitis are more likely to have low vitamin D levels. Notably, vitamin D supplementation has been associated with a significant reduction in uveitis activity. This finding further reinforces the crucial immunomodulatory role of vitamin D in the context of inflammatory eye diseases [21]. Although primarily a corneal rather than retinal disorder, a pilot study demonstrated that vitamin D supplementation might stabilize keratoconus progression. This effect was attributed to vitamin D’s capacity to modulate systemic inflammation and contribute to corneal structural stabilization. This example illustrates the broader protective role of vitamin D in ocular tissues, extending beyond the retina to other eye components [22].

## Challenges, Research Gaps, and Future Directions

Despite the compelling associations observed - particularly for diabetic retinopathy -findings from various studies remain somewhat inconsistent. These discrepancies can often be attributed to variations in study design, population characteristics, and methodologies used for measuring vitamin D levels and classifying DR severity [14].

The notable discrepancy between observational study findings and large-scale randomized controlled trials (as observed in AMD) highlights the inherent complexity and challenges involved in definitively establishing causality and therapeutic efficacy for nutritional interventions [14].

The gap between strong observational evidence and the often null or inconclusive results from RCTs (particularly evident for AMD and the recognized need for more RCTs for DR) highlights a significant translational gap in this field. This suggests that biological plausibility and observed statistical associations do not automatically translate into practical or universally applicable clinical interventions, underscoring the complexity and challenges inherent to nutritional intervention research [13].

Current scientific literature reveals a critical gap regarding the definitive therapeutic role of vitamin D supplementation in both preventing the onset and managing the progression of diabetic retinopathy. Therefore, there is an urgent need for more rigorous and extensive research, particularly large-scale longitudinal studies and randomized controlled trials, to establish a clearer causal relationship and to determine optimal dosing strategies for retinal health outcomes [13]. Despite its negative findings for AMD, the VITAL study provides invaluable insights into the methodological challenges and complexities of conducting large-scale supplementation trials for chronic, multifactorial diseases [1,4].

The exact optimal dose of vitamin D for various health outcomes, including retinal health, remains a subject of considerable scientific debate. While some studies suggest a general daily supplementation of 2000 IU/day for broader health benefits, and even higher doses of 4000-6000 IU/day for potentially greater protection against adverse health outcomes, the specific optimal doses tailored for retinal health or for managing retinal diseases have not yet been definitively established [13,23].

The “threshold effect” observed for DR risk - where risk rises significantly only when vitamin D levels fall below 20 ng/mL - suggests that simply preventing severe deficiency may be enough to mitigate DR risk, rather than necessarily aiming for very high serum concentrations. This finding provides a strong rationale for a more personalized approach to vitamin D assessment and supplementation, based on an individual’s current vitamin D status [18]. The variability in suggested optimal levels and, mainly, the identified threshold effect for DR risk, suggest that a one-size-fits-all approach to vitamin D supplementation may not be the most effective strategy for retinal health. Future research and clinical practice should increasingly focus on personalized strategies that consider individual vitamin D status, genetic predispositions, and specific retinal pathologies [1,3].

To advance understanding and clinical application, future research should delve deeper into the complex interplay between 1,25(OH)_2_D_3_ and membrane-acting agents, as well as conduct more detailed analyses of intercellular communication in the complex retinal microenvironment. Integrating these emerging molecular insights into clinical practice is essential for optimizing vitamin D’s overall impact on metabolic health and on retinal outcomes [6,24].

The explicit call for continued molecular research, alongside the demand for more robust clinical trials, underscores the ongoing and crucial need to bridge the gap between fundamental basic science understanding and practical clinical application [6].

A deeper and more comprehensive mechanistic understanding at the cellular and molecular levels can directly inform the development of more targeted, effective, and personalized therapeutic strategies for retinal diseases.

## Conclusion

### Vitamin D as a Modifiable Factor in Retinal Health

Vitamin D plays a diverse, complex, and undeniably critical role in maintaining the physiological integrity of the retina - a role that extends far beyond its classical functions in bone health. Its profound influence is fundamentally mediated by the widespread presence of the Vitamin D Receptor (VDR) in various retinal cell types and by the demonstrated capacity for local activation of vitamin D within ocular tissues themselves.

Its multifunctional actions, encompassing potent anti-inflammatory, antioxidant, anti-angiogenic, and neuroprotective properties, are vital for supporting the highly complex cellular environment, maintaining the delicate vascular network, and meeting the retina’s exceptionally high metabolic demands.

Compelling and consistent observational evidence, robust in the context of diabetic retinopathy, firmly suggests that vitamin D deficiency is a significant and, crucially, modifiable risk factor for various retinal pathologies. Therefore, strategically addressing vitamin D deficiency through appropriate supplementation could represent a novel and promising pathway to reduce the substantial burden of DR and potentially other retinal diseases.

While the current body of evidence is auspicious, the field unequivocally requires more robust, large-scale randomized clinical trials. These studies are essential for definitively establishing causal relationships, determining optimal dosing strategies for specific retinal conditions, and assessing the long-term effects of vitamin D supplementation on retinal health outcomes.

Furthermore, future research should increasingly focus on personalized approaches to vitamin D supplementation. This involves integrating an individual’s specific vitamin D status with their unique genetic predispositions and the characteristics of their particular retinal pathologies to optimize therapeutic interventions and ultimately improve overall retinal health.
